# Neonatal mature teratoma arising from an intra-abdominal cryptorchid testis with torsion: a case report and literature review

**DOI:** 10.3389/fped.2026.1802253

**Published:** 2026-04-10

**Authors:** Tong Liu, Shaoqing Guo, Yanfei Chen

**Affiliations:** 1 Department of Pediatrics, School of Medicine, The First Affiliated Hospital of Xiamen University, Xiamen University, Xiamen, Fujian, China; 2 Pediatric Key Laboratory of Xiamen, Xiamen, Fujian, China

**Keywords:** abdominal mass, alpha-fetoprotein, cryptorchidism, neonate, teratoma

## Abstract

**Introduction:**

This study investigated the clinical characteristics, diagnostic considerations, and optimal management of a neonatal mature teratoma arising from an intra-abdominal cryptorchid testis complicated by torsion, a rare clinical entity.

**Methods:**

We retrospectively analyzed data from a full-term male neonate admitted in November 2025 with a prenatally detected intra-abdominal mass, including postnatal imaging, tumor marker assessment, surgical excision, and pathological confirmation. Concurrently, we conducted a systematic review of English-language literature using PubMed, Embase, Cochrane Library, and Web of Science.

**Results:**

The patient was diagnosed with a mature teratoma originating from an intra-abdominal cryptorchid testis. Intraoperative findings revealed 360° clockwise torsion causing colonic compression. Complete resection resulted in an uneventful recovery, with a physiological decline in serum alpha-fetoprotein (AFP) levels consistent with postnatal age-related maturation, and no recurrence at one-month follow-up. The literature review identified ten cases (including the present case) of prenatally detected ultrasound-detected tumors with progressive tumor growth. Surgical management primarily involved laparoscopic surgery (40.0%) or laparotomy (60.0%). Mature teratomas were the predominant pathology (90.0%), tumor torsion occurred in 20.0% of cases, and no recurrences were reported, indicating a favorable prognosis.

**Conclusion:**

Neonatal mature teratomas arising from an intra-abdominal cryptorchid testis with torsion are exceedingly rare. Evaluation of testicular position should be routine in male neonates with intra-abdominal masses, and gonadal tumors should be included in the differential diagnosis. Multidisciplinary, individualized management is essential for optimizing outcomes.

## 1 Introduction

Childhood testicular tumors are rare, with an incidence rate ranging from 0.5 to 2.0 per 100,000 prepubertal males. These tumors account for 1%–2% of all childhood solid tumors and are predominantly unilateral (1, 2). Its incidence exhibits a bimodal distribution (3). The first peak occurs in infants under 3 years of age, where tumors are mainly benign, whereas the second peak arises after puberty and is characterized by a notable increase in malignant cases. Established risk factors include cryptorchidism, genetic abnormalities, and a family history of germ cell tumors (4, 5).

Teratomas are common solid tumors of the neonatal period and are typically located in the sacrococcygeal region or gonads. However, the development of a teratoma from an intra-abdominal cryptorchid testis is exceedingly rare, with an incidence of testicular teratoma in neonates of only 0.015 to 0.06 per 1 million (6, 7). Testicular teratoma torsion, a serious complication of this condition, has been reported in multiple cases across all age groups; however, reports specifically in the neonatal period remain exceedingly rare, with only a few documented cases (8).

## 2 Case description

### 2.1 Prenatal history and birth status

The infant's mother was a healthy 35-year-old, gravida 2, para 1, with no family history of genetic disorders. At 34 weeks of gestation, ultrasound revealed a heterogeneous echogenic mass in the left lower abdomen of the fetus, characterized by parallel rod-shaped strong echoes. This mass increased in size as the pregnancy advanced, with prenatal ultrasound measurements showing growth from 2.54 × 1.97 cm (two-dimensional) at 34 weeks to 4.2 × 2.9 × 4.5 cm (three-dimensional) on postnatal computed tomography (CT). The child was delivered vaginally at 39 weeks and 4 days, weighing 3.6 kg at birth, with Apgar scores of 10 at both 1 and 5 min. Postnatally, the infant had normal suckling and feeding ability, normal flatus and defecation, and no clinical signs of intestinal obstruction (no abdominal distension, vomiting, or failure to pass stool/flatus).

### 2.2 Neonatal assessment and diagnosis

#### 2.2.1 Physical examination

Vital signs were stable. A firm, well-demarcated mass was palpable in the left lower abdomen with limited mobility and no apparent tenderness. The left scrotum was empty, and the left testis was not palpable in the left inguinal region.

#### 2.2.2 Auxiliary tests

**Figure 1:**
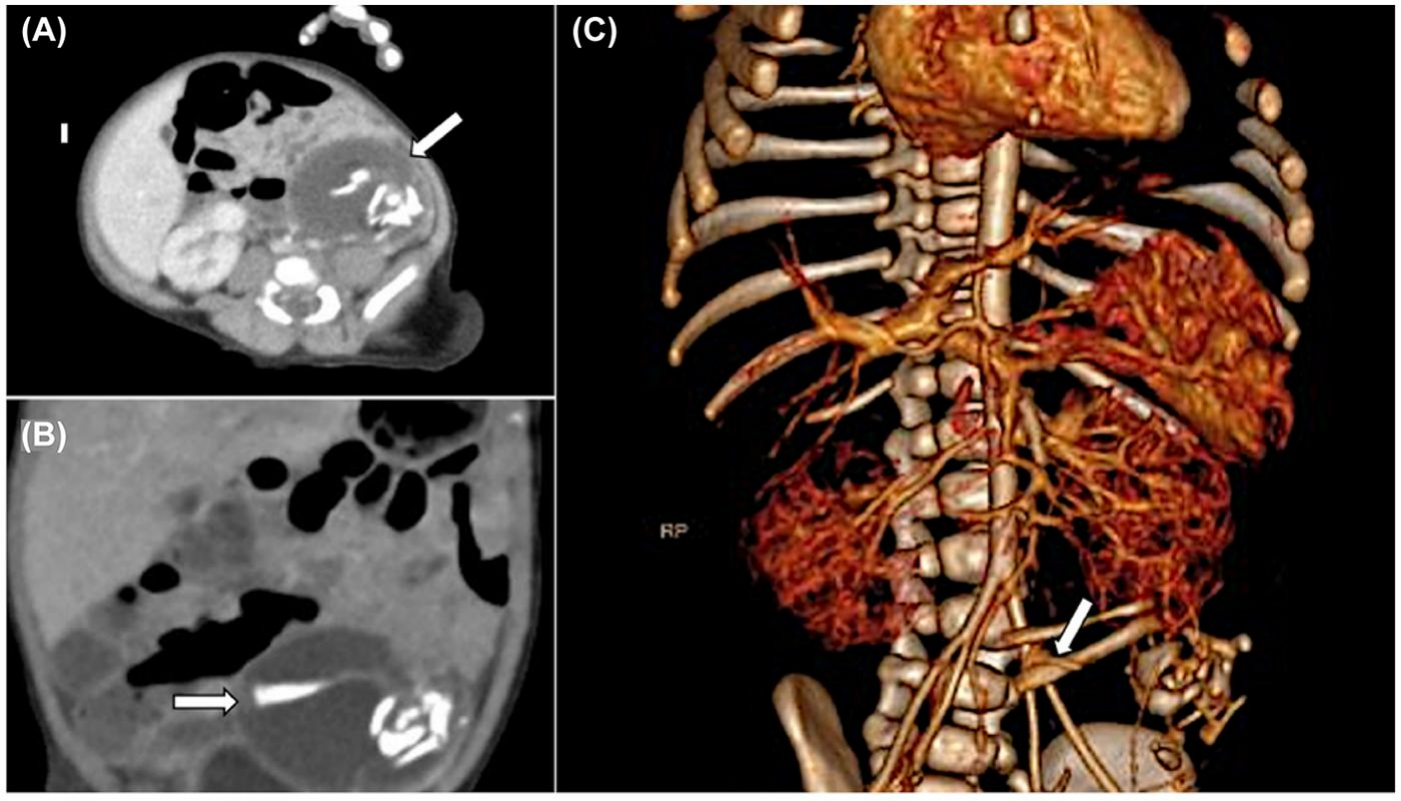
Abdominal CT images. **(A)** is a cross-section reveals a mixed-density space-occupying lesion in the lower left abdomen, marked by a white arrow. This lesion contains fat density shadows and patchy high-density bony structures. **(B)** is a coronal image. **(C)** is a three-dimensional reconstruction image of the tumor's pedicle is attached to the left iliac blood vessel.

Postnatal abdominal ultrasound revealed a 6.2 × 2.9 × 4.0 cm mixed echo mass in the left lower abdomen. Abdominal CT in Figure 1 (A: cross-section; B: coronal image; C: three-dimensional reconstruction) demonstrated a heterogeneous dense mass in the left middle and lower abdomen containing fat and bone-like structures, with no significant enhancement following contrast administration. The serum AFP level at 23 h after birth was 43,972.48 ng/mL, falling within the age-specific normal reference range for term neonates on postnatal day 1.

### 2.3 Treatment and surgery

**Figure 2:**
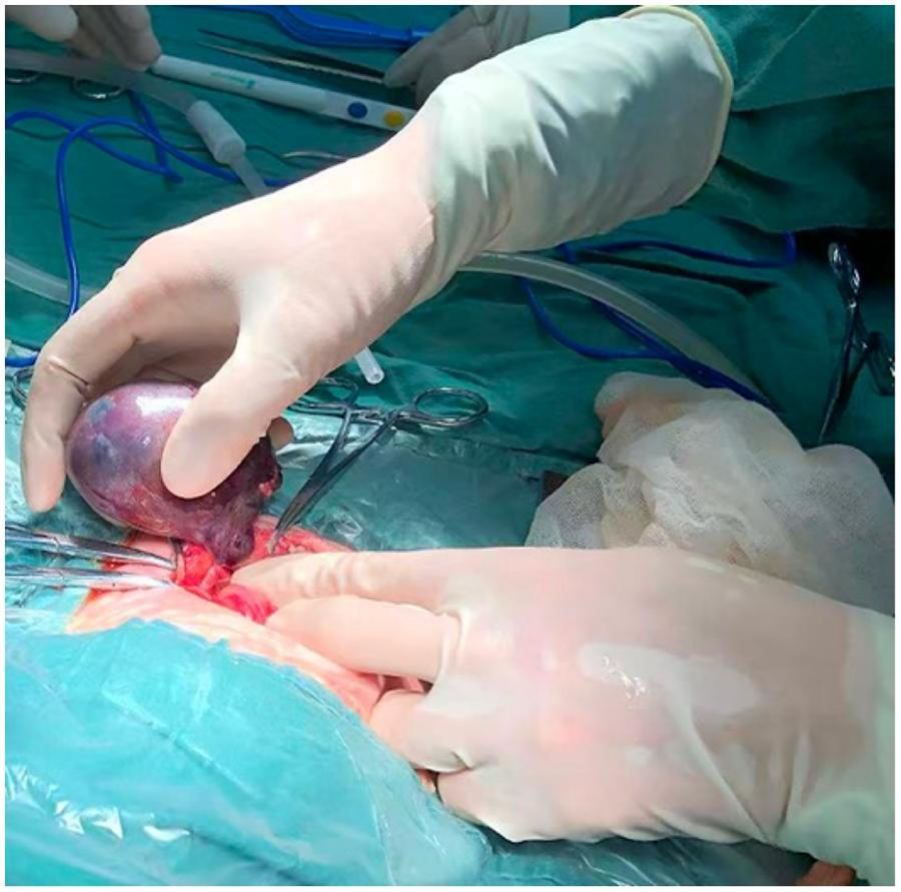
Intraoperative finding. Photograph during exploratory laparotomy shows the well-encapsulated, cystic-solid tumor with its pedicle twisted 360° clockwise.

Following a multidisciplinary consultation involving neonatology, pediatric surgery, radiology, and anesthesiology, the infant underwent exploratory laparotomy under general anesthesia on the tenth day post-birth. Intraoperative exploration revealed a 6 × 5 × 5 cm cystic-solid mass in the left lower abdomen and pelvic cavity (Figure 2), enclosed within a complete capsule. The mass was attached to the left iliac vascular region via a pedicle and underwent 360° clockwise torsion, causing compression of the colon. No left testis was identified during surgery. Complete resection of the tumor was successfully performed.

### 2.4 Pathological examination

#### 2.4.1 Gross specimen

**Figure 3:**
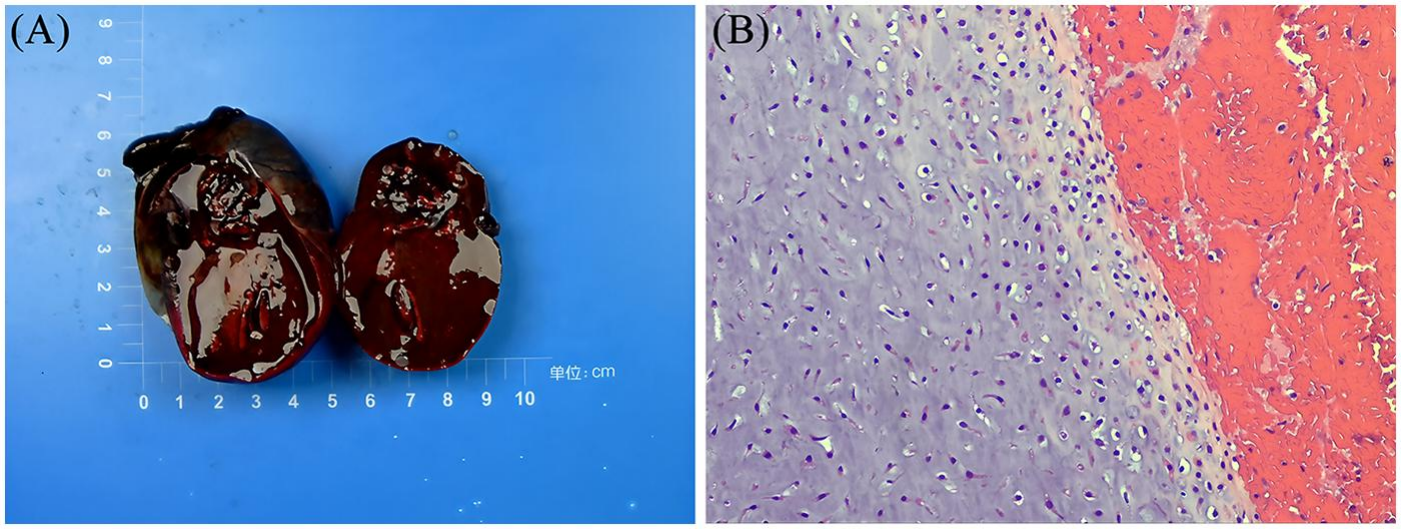
Pathology and histopathology of the teratoma. **(A)** shows the gross pathology of the resected specimen. **(B)** shows the histopathological features of the mature teratoma (Hematoxylin and Eosin staining).

The resected mass measured 6.7 × 5 × 4.3 cm (Figure 3A) and exhibited a smooth surface and an intact capsule. The cut surface was dark red and solid and contained visible bone tissue.

#### 2.4.2 Histopathological examination

Microscopic analysis revealed mature tissues derived from all three germ layers (Figure 3B), including stratified squamous epithelium, hair follicles, cartilage, bone, and intestinal mucosa, accompanied by stromal vascular congestion and focal hemorrhagic necrosis.

#### 2.4.3 Immunohistochemistry

Immunohistochemistry investigations revealed Steroidogenic Factor 1 (SF1) negative, Sal-like Protein 4 (SALL4) scattered positive, Octamer-Binding Transcription Factor 3/4 (OCT3/4) negative, and Cluster of Differentiation 117 (CD117) scattered positive.

#### 2.4.4 Pathologic diagnosis

The mass was diagnosed as a mature teratoma arising from an intra-abdominal cryptorchid testis.

### 2.5 Postoperative follow-up

Ten days after surgery, the serum AFP level decreased to 2,517.22 ng/mL. Postoperative ultrasonography revealed no testicular-like echoes in the left scrotum or inguinal region. The infant recovered uneventfully and was discharged on the tenth postoperative day. At the one-month outpatient follow-up, the infant was in good general condition with normal growth and development, and no signs of recurrence were observed.

## 3 Literature review

### 3.1 Literature search strategy

A systematic search was conducted in PubMed, Embase, Cochrane Library, and Web of Science databases from January 1, 1997, to December 31, 2025. The search strings utilized included “testicular tumour”, “undescended testis”, “intra-abdominal mass”, “neonate”, “newborn”, “infant”, and “teratoma”.

The inclusion criteria were as follows: (1) diagnosis within the neonatal period (≤28 days); (2) intra-abdominal tumor location; (3) pathologically confirmed teratoma originating from an undescended testis; and (4) case reports or series studies with complete clinical data.

The exclusion criteria were as follows: (1) age >28 days; (2) tumor located in the scrotum or inguinal region; (3) unclear pathological type; and (4) incomplete clinical data precluding the extraction of key information.

Ultimately, nine eligible articles were included, and a total of ten cases (including the current case) were analyzed.

### 3.2 Clinical characteristics of included cases

All ten infants were full-term neonates, with five cases of left-sided cryptorchidism and five cases of right-sided cryptorchidism. Abdominal masses were detected via prenatal ultrasound in all cases, and no specific clinical symptoms were observed during the neonatal period (Table 1). Tumor sizes ranged from 1.5 to 10.0 cm: six cases (60.0%) were <4 cm, three cases (30.0%) were 4–8 cm, and one case (10.0%) was >8 cm. Tumor torsion was present in two cases (20%). All tumors exhibited progressive growth. Imaging examinations (ultrasound, CT, or MRI) revealed mixed-density or mixed-echogenicity lesions, some of which displayed fat, calcification, or bone-like structures. Preoperative AFP levels varied: four cases (40%) had values above the general pediatric reference ranges (yet all were within age-specific normal ranges for term neonates), four cases (40%) had levels consistent with neonatal age-specific normal ranges, and AFP levels were not recorded for two cases (20%; Table 2). Notably, all AFP values in the included cases should be interpreted against the age-specific reference ranges for full-term neonates.

**Table 1:** Summary of clinical characteristics of teratomas originating from intraperitoneal cryptorchid testes in neonates (*n* = 10).

Clinical characteristics	Number of cases	Proportion (%)
Age		
Neonatal period (≤28 days)	10	100.0
Side of cryptorchidism		
Left	5	50.0
Right	5	50.0
Clinical manifestations		
Asymptomatic (prenatally detected)	10	100.0
Maximum tumor diameter		
<4 cm	6	60.0
4–8 cm	3	30.0
>8 cm	1	10.0
Tumor torsion	2	20%
Surgical methods		
Laparoscopic surgery	4	40.0
Laparotomy	6	60.0
Among which: testis-sparing surgery	1	10.0
Pathological diagnosis		
Mature teratoma	9	90.0
Immature teratoma	1	10.0
Follow-up and recurrence		
With follow-up data	7	70.0
Recurrence	0	0.0

**Table 2:** Detailed summary of neonatal teratomas originating from intra-abdominal cryptorchid testes.

Author	Gestational age at first detection (weeks)	Size (cm) and location of mass at detection	Gestational age at birth (weeks)	Preoperative examinations	Preoperative AFP	Timing of surgery (post-birth)	Surgical method	Tumor size during surgery (cm)	Tumor torsion	Pathological type	Follow-up duration	Recurrence
Mboyo et al. (9)	31	Left para-bladder, 2.5 × 2.3	Term	US	7,980 mg/mL	13 days	Left orchiectomy	10 × 7.5 × 3	No	MT	1 year	No
Shih et al. (10)	36	Anterior to right kidney and bladder, 5 × 4 × 3	Term	US	21,226 ng/mL	6 days	Right tumor resection	5 × 4 × 4	No	MT	1 year	No
Siu et al. (11)	30	Below liver, anterior to right kidney and above bladder, 2.4	38	US	Normal	1 month	Laparoscopic right testicular tumor resection	1.5	No	MT	/	No
Pramanik et al. (12)	27	Right iliac fossa, 2.1 × 1.9	Term	US/MRI	4.92 ng/mL	5 months	Intra-abdominal tumor resection	4.9 × 4.4 × 4	No	MT	/	No
Janda et al. (8)	22 + 1	Pelvic cavity, 1.01 × 2	37 ^+^ ^1^	US	/	19 days	Left tumor resection	1.8 × 1.3 × 1.4	Yes	MT	/	No
Youssef et al. (13)	32	Between left kidney and bladder, 2 × 2 × 2.2	37	US	/	3 days	Laparoscopic tumor resection	3	No	MT	1 year	No
Arkar et al. (14)	36	Left side of bladder, 2 × 1.8	Term	US	Normal	3 days	Intra-abdominal tumor resection	2 × 1.5	No	MT	/	No
Yada et al. (15)	33	Retroperitoneal right side, 3.8 × 2.8	38	US/CT	Normal	14 days	Laparoscopic tumor resection	/	No	IMT	3 years	No
Fang et al. (16)	28	Above right bladder, 1.6 × 1.1	39	US	473 ng/mL	1 month	Fowler–Stephens orchiectomy	2.8 × 1.8 × 1	No	MT	7 months	No
Current case	34	Left lower abdomen, 2.54 × 1.97	39 ^+^ ^4^	US/CT	43,927.48	10 days	Intra-abdominal tumor resection	6.7 × 5 × 4.3	Yes (360° clockwise)	MT	1 month	No

US, Ultrasound; CT, computed tomography; MRI, magnetic resonance imaging MT, mature teratoma; IMT, immature teratoma; “/”, indicates data not recorded in the literature.

### 3.3 Treatment and prognosis

All infants underwent surgical treatment, with the procedures performed between 3 days and 5 months postpartum. Of these, seven cases (70.0%) were operated on during the neonatal period. The surgical approaches included laparoscopic surgery in four cases (40.0%) and open abdominal surgery in six cases (60.0%). Fang et al. (16) reported that the tumor was small, and intraoperative frozen section analysis confirmed its benign nature, leading to an attempt at testis-sparing surgery. The remaining nine patients underwent complete tumor resection. Pathological diagnosis revealed nine cases (90.0%) of mature teratoma and one case (10.0%) of immature teratoma. Complete follow-up data were available for seven cases, with follow-up durations ranging from 1 month to 3 years, during which no tumor recurrence was observed (Tables 1, 2).

Table 2 presents detailed information on the ten cases from the included literature. Yada et al. (15) documented a case of an immature teratoma with no recurrence after a three-year postoperative follow-up. The other nine cases involved mature teratomas, with no tumor recurrence or metastasis observed during follow-up. Recently, laparoscopic surgery has become increasingly popular due to its minimally invasive nature and rapid recovery times (16). However, for tumors with large volume or complex anatomical location, laparotomy remains the primary surgical approach (17).

## 4 Discussion

Cryptorchidism is a common urogenital malformation in male neonates, affecting approximately 1%–5% of full-term infants (17). The risk of tumor development in cryptorchid testes is approximately 10%, with intra-abdominal cryptorchidism carrying a 4–6 times higher cancer risk than normally descended testes (18). Teratomas, which originate from primordial germ cells, contain differentiated tissues from all three germ layers and are classified as mature or immature based on their degree of differentiation (7). Most teratomas in neonates are mature and exhibit low malignancy, reflecting the high differentiation potential and low malignancy risk of germ cells in infancy (19). In this case, the tumor developed from an intra-abdominal cryptorchid testis, which is a high-risk scenario. Its pathogenesis may involve abnormal migration and differentiation of primordial germ cells (20).

The diagnosis of intra-abdominal testicular tumors often relies on imaging examinations. Prenatal ultrasound is crucial for early detection and is the preferred modality for evaluating abdominal masses in newborns (13). Teratomas typically appear on ultrasound as mixed cystic-solid masses, often with visible fat, calcification, or ossification foci (11). Abdominal CT or MRI can further define the tumor's anatomical location and size, assess its relationship with adjacent blood vessels and organs, and evaluate for distant metastasis, thereby aiding surgical planning (1).

Tumor markers such as AFP, *β*-HCG, and LDH are important for distinguishing benign from malignant germ cell tumors in older children and adults, and elevated serum AFP levels are closely associated with teratoma recurrence and malignant transformation (21). However, interpretation of AFP levels in neonates is challenging because physiological elevation with a wide fluctuating range is characteristic of the first postnatal year (22). The temporal trend of AFP decline may therefore serve as a more reliable biomarker for identifying malignant lesions and monitoring postoperative recurrence (23). Although *β*-HCG and LDH may provide additional information regarding tumor characteristics, their utility in the neonatal period remains limited. The definitive diagnosis depends on the pathology biopsy report.

In neonates, the detection of an abdominal mass concurrent with an empty ipsilateral scrotum is a key indicator of a tumor originating from the intra-abdominal cryptorchid testis. Given that most documented cases exhibit this characteristic, routine evaluation of testicular position in male neonates with intra-abdominal masses is imperative. Furthermore, differentiating this condition from other neonatal intra-abdominal tumors (24), such as neuroblastoma, yolk sac tumor, lymphangioma, and intestinal duplication cysts, is crucial.

Surgical resection is the preferred treatment modality, and early intervention is critical to prevent torsion and necrosis (25).

Surgical techniques included laparotomy and laparoscopy. Laparotomy is generally recommended when a tumor exceeds 3 cm in diameter or its nature remains indeterminate preoperatively (26). Conversely, laparoscopic surgery (15) offers advantages such as reduced trauma, a lower risk of postoperative adhesive intestinal obstruction, and accelerated recovery, rendering it suitable for smaller, superficially located tumors.

Recurrence or metastasis of testicular teratoma following orchiectomy or testis-sparing surgery (TSS) has been infrequently documented. Therefore, strict adherence to the established criteria for TSS (27, 28) is essential: the tumor must be small (typically <3 cm), exhibit well-defined margins, be confirmed as benign via intraoperative frozen-section pathology, demonstrate negative surgical margins, and the remaining testicular tissue must maintain normal function. Patients who meet these criteria require close monitoring during the first postoperative year. Among the ten neonatal cases of intra-abdominal cryptorchid testicular teratoma included in our systematic review (nine previously reported cases plus the present case), only one patient underwent TSS. In contrast, the remaining nine underwent complete tumor resection because the tumors were either large or tightly adherent to the cryptorchid tissue, precluding the preservation of normal testicular tissue.

In cases of immature teratomas, the risk of malignancy during the neonatal period is low. Following complete resection, the prognosis is favorable, and routine chemotherapy is typically not required (28); however, vigilant monitoring for recurrence is essential. Therefore, long-term postoperative follow-up is crucial for improving prognosis in children (25). This follow-up should include serum AFP measurements at 1, 3, 6, and 12 months post-surgery (interpreted against age-matched reference ranges); imaging examinations, such as abdominal ultrasounds at 3, 6, and 12 months; and assessments of reproductive health, including the development of the contralateral testis, secondary sexual characteristics, and the onset of puberty.

This study included a case report and a retrospective analysis of a small sample of ten cases. The absence of comprehensive follow-up data in some studies may introduce selection bias. Furthermore, genetic testing was not performed in this patient, precluding the identification of potentially relevant genetic factors. Future research should incorporate prospective studies with larger sample sizes to further explore the genetic mechanisms and long-term prognosis.

## 5 Conclusion

This case represents a rare instance of neonatal intra-abdominal cryptorchid testicular teratoma complicated by torsion. While testicular torsion as a complication of testicular teratoma has been reported in various age groups, such cases occurring in the neonatal period remain exceedingly rare, with only a limited number of documented reports in the literature. Notably, it comprehensively documents a 360° clockwise torsion and the resulting compression of adjacent organs, thereby enhancing our understanding of the complications linked to this condition. For male neonates presenting with abdominal masses, routine evaluation of testicular position is recommended, and gonadal-origin tumors should be considered in the differential diagnosis. Multidisciplinary collaboration is crucial to optimizing diagnosis and treatment, ultimately improving the prognosis of affected infants.

## 6 Patient perspective

As the parents of our newborn son, we learned about his abdominal mass during a routine 34-week prenatal ultrasound. This unexpected news filled us with anxiety and confusion; we knew nothing about this rare condition and feared for his health.

After his birth, a multidisciplinary team (neonatology, pediatric surgery, and radiology) clearly explained the diagnosis (an intra-abdominal cryptorchid testis-derived teratoma) and the need for early surgery. Their patience in answering our questions about torsion risks and recovery eased our fears, and the attentive care provided by the nurses during the 10-day preoperative observation period provided great comfort.

The surgery went smoothly, and we felt immense relief when we were informed that the tumor was fully resected and pathologically benign. Watching him recover steadily and bringing him home on postoperative day 10 brought indescribable joy. His healthy growth and absence of recurrence at the one-month follow-up filled us with deep gratitude.

To other families facing similar prenatal diagnoses: trust the medical team's expertise, participate in treatment decisions, and hold hope. We sincerely thank all healthcare providers for their skills, compassion, and dedication in safeguarding our babies' health.

## Data Availability

The original contributions presented in the study are included in the article/Supplementary Material, further inquiries can be directed to the corresponding author.
